# The Diverse Nature of the Molecular Interactions That Govern the COV-2 Variants’ Cell Receptor Affinity Ranking and Its Experimental Variability

**DOI:** 10.3390/ijms25052585

**Published:** 2024-02-23

**Authors:** Fredy Sussman, Daniel S. Villaverde

**Affiliations:** Department of Organic Chemistry, Faculty of Chemistry, Universidad de Santiago de Compostela, 15784 Santiago de Compostela, Spain; daniel.sussman@raii.usc.es

**Keywords:** COV-2, variants of concern, variant infectivity, spike–receptor affinities, molecular mechanism, molecular mechanics calculations

## Abstract

A critical determinant of infectivity and virulence of the most infectious and or lethal variants of concern (VOCs): Wild Type, Delta and Omicron is related to the binding interactions between the receptor-binding domain of the spike and its host receptor, the initial step in cell infection. It is of the utmost importance to understand how mutations of a viral strain, especially those that are in the viral spike, affect the resulting infectivity of the emerging VOC, knowledge that could help us understand the variant virulence and inform the therapies applied or the vaccines developed. For this sake, we have applied a battery of computational protocols of increasing complexity to the calculation of the spike binding affinity for three variants of concern to the ACE2 cell receptor. The results clearly illustrate that the attachment of the spikes of the Delta and Omicron variants to the receptor originates through different molecular interaction mechanisms. All our protocols unanimously predict that the Delta variant has the highest receptor-binding affinity, while the Omicron variant displays a substantial variability in the binding affinity of the spike that relates to the structural plasticity of the Omicron spike–receptor complex. We suggest that the latter result could explain (at least in part) the variability of the in vitro binding results for this VOC and has led us to suggest a reason for the lower virulence of the Omicron variant as compared to earlier strains. Several hypotheses have been developed around this subject.

## 1. Introduction

Severe acute respiratory syndrome coronavirus 2 (SARS-CoV-2) is the causative agent of COVID-19, which was first reported in Wuhan (China) at the end of 2019 and which escalated into a global pandemic in 2020, producing, to date, hundreds of millions of infections and more than 6,000,000 deaths besides the countless human, social and economic disruptions, according to the World Health Organization (WHO). SARS-CoV-2 continuously undergoes mutations due to changes in the genetic code that usually occur during the replication of its genome. The new strains go unchecked because the virus lacks proofreading machinery. In the case of SARS-CoV-2, the mutants that have spread more widely and produced a larger number of deaths have been named variants of concern (VOCs) by the WHO. The ones that have proven to have the most lasting effects are the Delta [1] and Omicron [2] VOCs and their variants.

The SARS-CoV-2 viral membrane has a transmembrane glycoprotein, the spike (S) glycoprotein, and the envelope protein, and it surrounds the flexible helical nucleocapsid. The spike glycoprotein engages human angiotensin-converting enzyme (ACE2) through the spikes that protrude from the virion and give it its characteristic crown shape. The interaction between the virus receptor and the cell allows for viral genetic material to be delivered to the host cell cytoplasm for replication [3].

**Figure 1:**
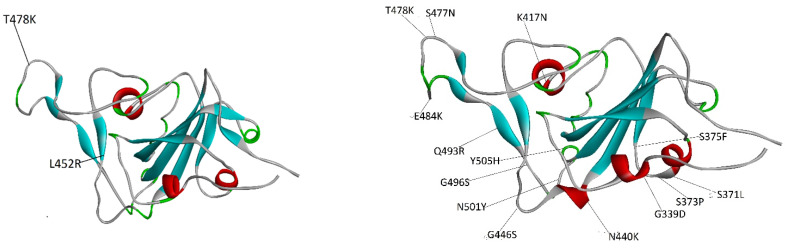
Placement of the mutations in the RBD spike of the Delta variant (**left-hand side**) and the Omicron variant (**right-hand side**). The Delta RBD has only 2 mutations whereas the Omicron RBD has 15 mutations. In the case of Delta, one mutation is in a loop (T478K) and the other is in a sheet (L452R). For its part, in Omicron, most of the mutations are in loops and sheets.

The segment of the spike that enters in direct contact with the ACE2 cell receptor is called the receptor-binding domain (RBD) and it contains many of the mutations that distinguish one VOC from others. Figure 1 displays the Delta and Omicron RBDs with the residue variants with respect to the native strain.

### 1.1. Hypothesis for Infectivity and Virulence of the VOCs

It is of the utmost importance to understand how mutations of a viral strain, especially those that are in the viral spike, affect the resulting infectivity of the emerging VOC, knowledge that could help us understand the variant virulence and inform the therapies applied or the vaccines developed. Several hypotheses have been developed around this subject.

#### 1.1.1. Spike–ACE2 Receptor Interactions

Since the initial step in cell infection is the viral cell entry that starts with the interaction of the spike trimer or monomer with the cell ACE2 receptor, it has been thought that this could be a rate-limiting step for viral entry into the cell [4,5,6,7] and hence an increase in the spike–ACE2 receptor affinity for the different VOCs could translate into enhanced infectivity. It has been observed that a greater infectivity does not always lead to enhanced virulence and lethality. Even though the Omicron variant has shown to be very infectious, it causes milder symptoms than previous VOCs, like the Wild Type and Delta, since it infects mostly the respiratory tracts rather than affecting the lungs.

The experimental spike–receptor affinities for a variety of mutant strains have been obtained independently by various research groups through Surface Plasmon Resonance (SPR) experiments [4,7,8] and microscale thermophoresis [9]. The results are shown in Table 1. 

As seen from this table, the results from different labs are dissimilar in many aspects. While the results of Han et al. indicate that the spike–receptor affinities for the Wild Type and Delta variants are very similar [7], the results of other labs show an increase in affinity for the latter strain (Delta). Moreover, only the results of the Im lab indicate that the larger number of mutations in the Omicron variant produces an increase in affinity [9]. Understanding the origin of the variability seen in these experiments is a very important step in determining the importance of the spike–receptor interaction (the first step in the virus cell entrance) in these VOCs’ infectivity.

#### 1.1.2. Spike–Antibody Interactions

An alternative source of increased infectivity upon the emergence of a new VOC is the ability of the new strain to reduce the affinity of the viral spikes for the antibodies produced by the organism and hence evade the immune system, as proposed for the omicron variant [4]. This knowledge should inform the design of antibody-based vaccines [10,11,12,13].

There has been an intensive effort aimed at understanding the spike–cell receptor and spike–antibody interactions using computational chemistry and especially molecular mechanics/molecular dynamics (MM/MD) tools [14,15,16,17,18,19,20,21,22,23,24,25,26,27]. The study of protein associations by MM/MD calculations represents a grand challenge in computational biochemistry since they involve the study of protein–protein interactions (PPIs), a difficult task given the size of the systems involved and the fact that protein–protein interactions (like antibody–antigen) are mediated by residue residents in loops, the most flexible secondary structure [27,28]. As seen from Figure 1, the spike interface with the receptor is predominantly made up of loop segments. Hence, most of the mutations of the Delta and Omicron variants reside in these segments. Given the flexibility, an exponential explosion of conformations should be expected, leading to sizable standard deviations in the calculation of binding affinities from MD simulations, which may exceed the average value of this property. Some of the authors have sidestepped this problem by calculating the binding affinities from the average over those frames that have the lowest binding energy [24]. In most of the calculations, the spike RBD–receptor complexes for the latter strains (Delta, Omicron) were modeled from the structure obtained by mutating the Wild Type strain (PDB entry 6m0j [29]) in silico, rather than starting from the actual experimental structures for the spike–receptor structures, a feature of the calculations that may have influenced the results. Since then, cryo-EM-based structures of the VOC spike RBD with the receptor are now available in the case of the Delta variant (PDB entry 7tew [30]) and in more than one case for the Omicron mutant (PDB entries 7u0n, 7wpb, 7wbp, 7t9l) [4,6,30,31,32]. The present work uses these experimentally determined structures, a route that will allow us to avoid the uncertainty of starting from modeled structures. 

Most previous work aiming at ranking the spike–receptor affinities of the VOCs studied in this work resulted in the following order (see, for instance, refs. [20,21,24]).

Native < Delta < Omicron. 

Sometimes, this was with sizable differences favoring the Omicron variant [20].

In this work, we propose a few protocols (some of them MD-based) aimed at predicting the affinities of any COV-2 RBD spike variant for the cell receptor. The methodology used here was designed to substantially reduce computer expense and hence can be applied to a wider range of larger systems describing the viral cell entry, without having to delve into coarse-grained approaches used previously [17]. The binding affinity prediction protocols have been organized as a battery of techniques of increasing complexity to evaluate the spike–receptor affinity. This strategy is expected to eliminate the bias from a single protocol and allow us to look for possible consensus amongst the predictions provided by these methods. As shown below, it has allowed us to determine that the Delta and Omicron variants’ RBD spikes enhance their ACE2 receptor affinity through very different molecular interactions. Our results have also allowed us to put forward an explanation for the variability observed in different SPR binding affinity experiments shown in Table 1.

## 2. Results and Discussion

### 2.1. Ranking Affinity Prediction by a Suite of Protocols of Increasing Complexity 

The most basic protocol for evaluating protein–protein affinities is related to the calculation of the surface area that is buried upon protein association, called Buried Surface Area (BSA) (For a review, see ref. [33]). We present the BSA-based affinity values from the six known PDB structures (see Table 2) as well as the average values generated by the spike–receptor association, obtained from the MD trajectories (see the Section 3 for details) for the three VOCs studied here (see Table 3). As shown from both types of calculations, the Delta variant is predicted to increase the binding affinity with respect to the original (Wild Type) strain, in line with the experiments of Mannar et al. [4] and Kim et al. [9]. On the other hand, this protocol predicts that the large number of mutations present in Omicron does not translate into a rise in affinity above the native variant. 

The above protein–protein interaction energy BSA-based protocol neglects many components of the binding free energy or at best only includes them indirectly. That is the case of the electrostatic interaction energy, which comprises both charge–charge interactions and the process leading to the desolvation of the charged groups during the binding process, as well as the contribution of entropy to the binding affinity. 

There have been many efforts in developing protocols that include all the relevant components of the protein–protein binding free energy. One of them is based on chemical thermodynamics principles and used for the automatic detection of macromolecular assemblies in the Protein Data Bank (PDB) entries in the PISA server [34]. Here, we have applied this method to all known spike RBD–receptor complexes. They include the Wild Type and Delta variants and the four Omicron structures (PDB entries 7t9l, 7u0n, 7wpb and 7wbp). The evaluation of these latter structures will allow us to determine how the structural variability of the loops where mutations reside (see Figure 1) affects the binding energy results. The results are shown in Table 4. 

Perusal of this table indicates that (once again) the spike RBD from the Delta variant is the one that is predicted to have the highest affinity for the ACE2 receptor. For some of the Omicron structures, their association energies are comparable to the ones obtained by Delta (as in the case of the 7t9l and 7wbp), but other Omicron structures like 7wpb and 7u0n present binding energies lower than even the native strain. 

To determine the possibility of interconversion of one structure into the others, we performed MD studies using the protocol described in the Section 3 for Omicron structures 7u0n and 7t9l as well as the Delta (7tew) and Wuhan (6m0j) structures. The binding energies were obtained using the molecular mechanics energy differences supplemented by the corresponding changes in implicit generalized Born solvation energy upon binding, performed on frames that resulted from extensive MD simulations (see the Section 3 for details). The binding affinities that resulted from these calculations are listed in Table 5. 

As seen from Table 5, the Delta variant is (once again) the mutant with the highest affinity, in agreement with all previous protocols employed here (see Table 2, Table 3 and Table 4). Moreover, this variant has the largest standard deviation (4.3 Kcal/mol), an outcome that has implications for the ranking of the Omicron spike binding whose evaluation started from the 7t9l structure. Although the spike mean binding energy from the latter structure is more positive than the average from Delta, their combined standard deviations make the Omicron binding energy results indistinguishable from those for the Delta strain.

The results displayed in Table 1, Table 2, Table 3, Table 4 and Table 5 support some of the conclusions reached by Mannar et al. [4] rather than the ones by Han et al. [7] or Kim et al. [9]. The mutations that give rise to the Delta variant (T478K and L452R) are definitely the ones that enhance the binding affinity of the viral spike for the receptor. The Omicron variant shares a mutation (T478K) with the Delta variant, but that feature is not enough to increase the Omicron spike–receptor affinity above the one found for the Delta variant, and in some cases not even above the native variant (see Table 2, Table 3 and Table 4). This outcome would imply that the couple of mutations present in the RBD of the Delta variant (T478K and L452R) indeed increase the affinity of the spike for the receptor. The surplus of mutations present in the Omicron variant (over the Delta one) does not seem to increase the binding affinity, a result obtained previously within the PISA prediction approach. This outcome is probably due to the fact that the contributions of single mutations seem to cancel each other out, a result that is backed up by high-throughput single-mutation assays [4,7]. Hence, it would seem that the large number of mutations present in the RBD of the Omicron spike is not designed to increase its affinity for the receptor but rather fulfill some other tasks like reducing the affinity of the antibodies for them, a feature that sets the Omicron mutant apart from other VOCs [4]. 

### 2.2. Affinity Ranking and Structural Variability

The variability in the ranking of the binding affinities for the native, Delta and Omicron (see Table 1) variants has been attributed by other authors to the differences in the experimental setup of these labs [9]. Our multi-protocol approach for the evaluation of the binding affinity of the spike RBD for its cognate receptor allowed us to reach the consensus conclusion that the Delta variant RBD consistently has the highest affinity amongst all VOCs, in all evaluations. The only outstanding issue relates to the affinity of the spike of the Omicron variant. The variability of the calculated spike–receptor binding energies (that have, as a starting point, structures obtained in different labs) may be puzzling at first sight. The superimpositions of any two Omicron structures indicate that their maximum RMS value hovers around 1.0 Å, a rather small value. This issue draws attention to the structural plasticity of the PPIs, especially the most variable regions. The rationale for this variability in the binding affinities results in vitro and in silico could be related to the sizable extent of the protein–protein interface. This feature may lead to different predicted binding receptor affinities even for the same spike strain for different starting structures. It may even lead to different binding poses (for the spike RBD). In this study, we have observed this trend for the Omicron variant. The protein–protein interface areas for the original PDB Omicron structures display different values (see Table 2). 

**Figure 2:**
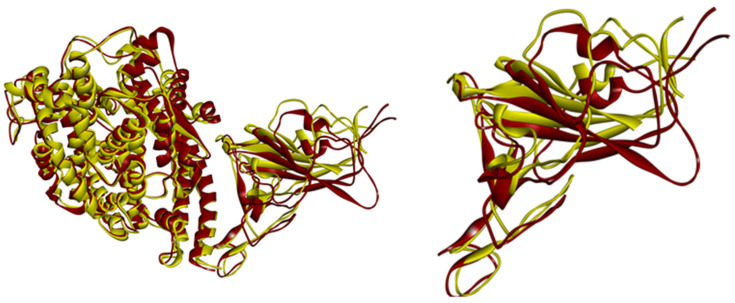
Overlap of the PDB structures 7u0n (yellow) and 7t9l (red). On the left panel is the full complex and on the right are the spike RBDs for both molecules. Notice the change in structure especially in the spike regions that are spanned by loops and sheets.

Still, the binding energy predictions for these two structures are quite different when a full MM/GBSA protocol is applied. To understand the structural underpinnings of this difference, we have superimposed the 7t9l and 7u0n structures (see Figure 2). In spite of the small RMS between these two structures (1.1 Ǻ), there are some noticeable differences between these two structures, more evident in the spike component, which has a preponderance of loops and beta sheets, whose conformation differs among these structures. 

One feature that sets the Omicron mutant apart from the earlier variants (Delta and native) is the very strong difference in the cell type tropism of the former type. While it is known that the native and Delta strains have a broad cell tropism that directly impacts into substantial viral replication rates in many tissues, the Omicron variant shows more restricted cell preferences. Its replication rates are similar for Delta in human nasal epithelial cultures and kidney cells [35] although it decreases substantially in gut cells and more importantly for lung cells [36].

The differences in replication were mapped to the entry efficiency of the virus on the basis of spike-pseudotyped virus assays [35]. Various rationales have been put forward to explain these differences including the lower efficiency of the S1/S2 cleavage of the Omicron variant by the TMPRSS2 protease [37].

In the present work, our results indicate that the first step in cell entry (that is, the spike–cell receptor interaction) is governed by a different molecular interaction mechanism for the Omicron mutant than for the native and Delta strains. Possibly, as a result, our calculations indicate a broad range of affinities of the Omicron spike for the receptor that varies from below the one found for the native strain to values that are close to the Delta mutant. It has occurred to us that these results could have a direct bearing on the Omicron cell type tropism. It could happen that in the lung tissue environment, the spike–receptor affinities have lower affinities than in some in vitro experiments, possibly lower than the ones for the native variant. This outcome could explain the low virulence of the Omicron viral type.

The results obtained here have allowed us to obtain insights into the molecular interactions that underpin the VOC binding affinities for the receptor and hence their binding ranking. As seen from Table 2 and Table 3, the results from the BSA-based affinity predictors clearly single out the Delta spike variant as the one with the higher affinity (over the native strain). Since the contact area calculated from the BSA should be based primarily on Van der Waals interactions, we expect that these are paramount for the increased affinity of the Delta strain. Only when electrostatic interactions and the corresponding desolvation terms are included in the calculations does the Omicron spike affinity for the receptor reach values close to that of the Delta spike (see Table 4 and Table 5). This outcome indicates that diverse VOCs’ spikes may increase their affinity over the native variant through different molecular interactions.

One outstanding issue is how the mutations found in the Delta variant modify the BSA due to spike–receptor interactions. Saville et al. argued that these residue modifications increase electrostatic complementarity between both proteins [30]. Perusal of the structure indicates indeed that new hydrogen bonds in the region of residues 500–505 of the spike are created in the Delta variant [30]. The mutants that define the Delta variant (T478K and L452R) lie far away from the above segment, so a question arises as to how they could produce this effect. 

**Figure 3:**
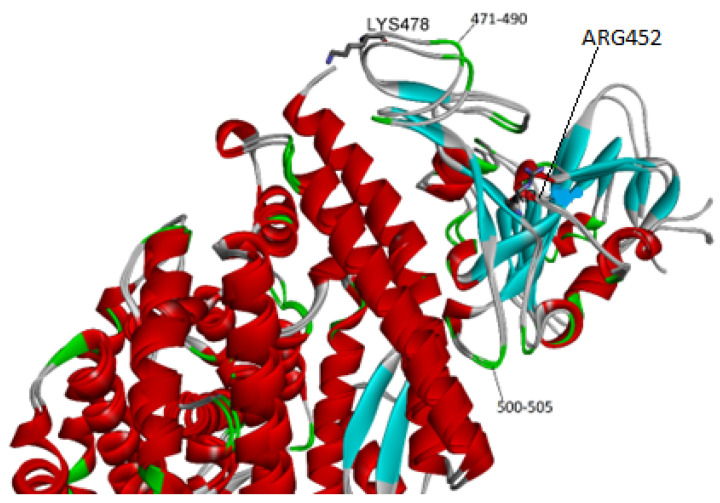
Close-up of the spike–receptor interface for the native (PDB entry 6m0j) and the Delta strain (PDB entry 7tew). Notice that the mutations (T478K and L452R) are not close to the receptor.

Overlap of the protein–protein complex for the native and Delta structures (see Figure 3) sheds some light. As expected, there are some changes in the segment that spans from 500 to 505 in the RBD. Nevertheless, there are wider differences in the backbone of the two loops (see residues 471–490) that are closer to Lys 478, one of two mutated residues. A possible explanation is that the latter residue exerts a pull effect that changes the location of the adjacent loops, resulting in an enhanced buried surface and binding affinity. The ACE2 cell receptor has many acidic residues and hence a highly negative charge (−23) that could produce an electrostatic attraction on Lys 478 that results in the loop drag mentioned above. 

## 3. Materials and Methods

All the structures used in this work were downloaded from the Protein Data Bank (PDB). The structures used in this study were the native (6m0j), the Delta (7tew) and the four Omicron structures obtained independently (7u0n, 7t9l, 7twbp and 7wpb. For the molecular mechanics-based studies, we only used the 7u0n and 7t9l structures for the Omicron studies. The input files for the molecular dynamics simulations were generated by the CHARMM-GUI Web interface [38]. All water and sugar molecules were discarded. The number of residues in each of the PDB structures differs slightly at the N and C terminals, so we took a template of the spike RBD and the ACE2 receptor, the one found in the native structure, and modified the other structures accordingly. The force field used in all simulations was CHARMM_36 [39]. To avoid the use of all atom solvents, we used the generalized Born with switching (GBSW) implicit solvation function [40]. In order to mimic the friction provided by the solvent, we used Langevin dynamics [41] with a friction factor (fbeta) of 20. In all cases, the time step was 2 femtoseconds, and a cutoff of 18 Ǻ was used for non-bonded interactions. The dynamics protocol was divided into two stages: In the first one, a dynamic trajectory of 2.5 ns was performed with initial heating, equilibration and preliminary production steps that added up to 0.5 ns, followed by ten steps, each one 0.2 ns. The structures saved after each of these steps were cooled down to 50K in two stages. The final frame of each trajectory (a total of 10) was saved for analysis of the spike–receptor interaction energies, which was evaluated using the same MM/GBSW protocol as the dynamics runs. 

Two other alternative evaluation protocols were applied. The simplest is based on the calculation of the Buried Surface Area (BSA) generated by the protein–protein association. This quantity has been shown to be a primary descriptor of the binding affinity or interaction energy of two macromolecular entities. A first-order approximation assumes that the BSAs could be proportional to the binding energies with a proportionality constant of 0.025 Kcal/mol per Å^2^ of surface protein removed from contact with water [33]. We applied this protocol to both the original spike–receptor structures found in the PDB, as well as to the frames that resulted from the last stage of our MD protocol, using the tools present in the graphical interface from Discovery Studio visualizer [42]. To find the accessible surface, we used a sphere the size of a water molecule (1.4 Å). The last and more elaborated protocol used here is based on a chemical thermodynamics approach which includes explicit entropic contributions to the protein–protein affinity [34]. 

### Associated Method Information

The input files for the MM/MD simulations (which included the parameter, connectivity files, coordinate files, etc.) were obtained through the input generator option of the CHARMM-GUI interface https://www.charmm-gui.org/. Last accessed on the 10 December 2023.

The MM/MD calculations were carried out by CHARMM version 46. The software can be found at https://www.charmm.org/archive/charmm/showcase/news/free-charmm/ and the documentation at https://www.charmm.org/archive/charmm/documentation. Last accessed 12 December 2023.

The parameter files and connectivity library can be found at https://github.com/fsussman/TOPPAR1.

The binding free energy calculations based on the Buried Surface Area (BSA) approach were performed on the PDB structures on the PISA-PDB server https://www.ebi.ac.uk/pdbe/pisa/. Documentation is available at the site. Last accessed on 5 November 2023.

The binding free energy calculations based on the BSA approach for the frames resulting from the MD trajectories were performed with the free version of Discovery Studio V3.5, which can be found at https://discover.3ds.com/discovery-studio-visualizer-download?gclid=CjwKCAjwu4WoBhBkEiwAojNdXpgnprVjkxLBhnSIdL1mRc6-7_twnRb26LNEP0y1-2G68t65nrpqSRoCH2wQAvD_BwE, Last accessed on 1 January 2023. Documentation is available for every task in the graphic interface.

## 4. Conclusions

The initial step in the COV-2 cell infection process (and possibly the rate-limiting step) is the interaction of the viral spike with its cognate receptor, the ACE2 enzyme. 

In this work, we have applied a battery of protocols of increasing complexity to the calculation of the spike binding affinity for three variants of concern to the ACE2 cell receptor. The results clearly illustrate that binding to the receptor (of the Delta and Omicron VOC spikes) occurs through different kinds of molecular interactions. While the Delta variant enhances its binding affinity above the one found for the native by an increased contact area between the proteins involved and hence through augmented Van der Waal contacts, the Omicron variant reaches the highest affinity by increasing electrostatic and entropic contributions.

The existence of various structures of Omicron complexes obtained by cryo-EM has allowed us to determine the effect of structural variability on the results. The results obtained indicate that there is a high variability in the binding affinity of the Omicron spike depending on the starting cryo-EM structures used. The highest binding affinity reaches at most the affinity value of the Delta variant, indicating that the surplus of mutations in the Omicron RBD segment of the spike exists to fulfill a different role possibly related to antibody escape [4]. Other cryo-EM structures produce binding affinities that are even lower than those calculated for the native strain (see Table 5), indicating that under some conditions, the Omicron spike may be a weaker binder than the native variant. The experimental results used here (see Table 1) were obtained in vitro. We have thought that the results obtained here could give some clue about the in vivo infectivity and lethality. It is known that the Omicron strain binds to the respiratory tract rather than to the lungs, a result that explains the high infectivity but low virulence of this variant. Our results indicate that the Omicron spike structure is endowed with a high variability and that some of the possible structures may bind to lung ACE2 receptors with an affinity predicted to be lower than the one calculated for the native variant spike, a result that could explain the lower virulence of this strain. 

The protocols presented here for the evaluation of the spike–receptor affinities involve substantial computer time savings within all atom molecular mechanics approaches for protein–protein interactions and will be especially useful for research in this field that requires intensive computer resources. One of the research lines we are involved in is the antibody evasion by some of the COV-2 VOCs. This is a major computational endeavor given the number of known antibodies, each one targeting different epitopes within the spike [43]. 

## Figures and Tables

**Table 1:** Spike–receptor affinities from various laboratories.

Source	Wild Type	Delta	Omicron
Mannar et al. (kd, nM) [4]	3.0 ± 0.3	2.0 ± 0.2	2.1 ± 0.3
Kim et al. (Kd, nM) [9]	27.5 ± 4.8	21.5 ± 2.9	5.5 ± 1.4
Han et al. (Kd, nM) [7]	24.63 ± 5.00	25.07 ± 6.70	31.40 ± 11.62
Zhang et al. (Kd, nM) [8]	------	2.9	8.9

**Table 2:** Spike–receptor association energies from Buried Surface Area calculations.

VOC (PDB Entry)	BSA ^(a)^	Binding Energy ^(b)^
Wild Type (6m0j)	−1719.2	−43.0
Omicron (7u0n)	−1720.6	−43.0
Omicron (7wbp)	−1733.1	−43.3
Omicron (7wpb)	−1710.7	−42.8
Omicron (7t9l)	−1678.2	−42.0
Delta (7tew)	−1807.6	−45.2

^(a)^ BSA values in Å^2 (b)^ energies in Kcal/mol.

**Table 3:** Spike–receptor association energies from MD frames on a BSA approach.

VOC/PDB	BSA ^(c)^	Binding Energy ^(a)^
Wild Type (6M0J)	−1750.9 (32.0) ^(b)^	−43.7 (0.8) ^(b)^
Delta (7TEW)	−1849.4 (19.6)	−46.2 (0.5)
Omicron (7T9L)	−1708.0 (48.0)	−42.7 (1.2)
Omicron (7U0N)	−1682.5 (50.9)	−42.1(1.3)

^(a)^ These are the average binding energies from the MD trajectories in Kcal/mol. ^(b)^ The number in parentheses is the standard deviation from the average of the property. ^(c)^ BSA values in Å^2^.

**Table 4:** Results for the binding of the RBD spike segment to cell receptor using PISA.

VOC	PDB Entry ^(b)^	Binding Energy (Pisa) ^(a)^
Wild Type	6m0j	−5.1
Delta	7tew	−6.3
Omicron	7t9l	−6.1
Omicron	7wpb	−3.4
Omicron	7u0n	−4.4
Omicron	7wbp	−6.2

^(a)^ Results in Kcal/mol, ^(b)^ calculations were performed directly from PDB structures.

**Table 5:** Binding energies from MD/GBSW protocol on MD trajectories.

VOC	PDB Entry	Binding Energy ^(a)^
Native	6m0j	−76.7(1.4) ^(b)^
Delta	7tew	−87.3 (4.3)
Omicron	7u0n	−67.0 (2.0)
Omicron	7t9l	−83.9 (1.0)

^(a)^ Energies in Kcal/mol. ^(b)^ Numbers in parentheses are standard deviations.

## Data Availability

Data is contained within the article.

## References

[1] Mlcochova P., Kemp S., Dhar M.S., Papa G., Meng B., Mishra S., Whittaker C., Mellan T., Ferreira I., Datir R. (2021). SARS-CoV-2 B.1.617.2 Delta variant replication, sensitivity to neutralising antibodies and vaccine breakthrough. Nature.

[2] Viana R., Moyo S., Amoako D.G., Tegally H., Scheepers C., Althaus C.L., Anyaneji U.J., Bester P.A., Boni M.F., Chand M. (2022). Rapid Epidemic Expansion of the SARS-CoV-2 Omicron Variant in Southern Africa. Nature.

[3] Jackson C.B., Farzan M., Chen B., Choe H. (2022). Mechanisms of SARS-CoV-2 entry into cells. Nat. Rev. Mol. Cell Biol..

[4] Mannar D., Saville J.W., Zhu X., Srivastava S.S., Berezuk A.M., Tuttle K.S., Marquez A.C., Sekirov I., Subramaniam S. (2022). SARS-CoV-2 Omicron variant: Antibody evasion and cryo-EM structure of spike protein-ACE2 complex. Science.

[5] Wrapp D., Wang N., Corbett K.S., Goldsmith J.A., Hsieh C.-L., Abiona O., Graham B.S., McLellan J.S. (2020). Cryo-EM structure of the 2019-nCoV spike in the prefusion conformation. Science.

[6] Han P., Li L., Liu S., Wang Q., Zhang D., Xu Z., Han P., Li X., Peng Q., Su C. (2022). Receptor binding and complex structures of human ACE2 to spike RBD from omicron and delta SARS-CoV-2. Cell.

[7] Starr T.N., Greaney A.J., Hilton S.K., Ellis D., Crawford K.H., Dingens A.S., Navarro M.J., Bowen J.E., Tortorici M.A., Walls A.C. (2020). Deep Mutational Scanning of SARS-CoV-2 Receptor Binding Domain Reveals Constraints on Folding and ACE2 Binding. Cell.

[8] Zhang X., Wu S., Wu B., Yang Q., Chen A., Li Y., Zhang Y., Pan T., Zhang H., He X. (2021). SARS-CoV-2 Omicron strain exhibits potent capabilities for immune evasion and viral entrance. Signal Transduct. Target. Ther..

[9] Kim S., Liu Y., Ziarnik M., Cao Y., Zhang X.F., Im W. (2023). Binding of human ACE2 and RBD of omicron enhanced by unique interaction patterns among SARS-CoV-2 variants of concern. J. Comp. Chem..

[10] Jangra S., Ye C., Rathnasinghe R., Stadlbauer D., Krammer F., Simon V., Martinez-Sobrido L., Garcia-Sastre A., Schotsaert M. (2021). The E484K mutation in the SARS-CoV-2 spike protein reduces but does not abolish neutralizing activity of human convalescent and post-vaccination sera. MedRxiv.

[11] Greaney A.J., Starr T.N., Gilchuk P., Zost S.J., Binshtein E., Loes A.N., Hilton S.K., Huddleston J., Eguia R., Crawford K.H.D. (2021). Complete Mapping of Mutations to the SARS-CoV-2 Spike Receptor-Binding Domain that Escape Antibody Recognition. Cell Host Microbe.

[12] Liu Z., VanBlargan L.A., Bloyet L.-M., Rothlauf P.W., Chen R.E., Stumpf S., Zhao H., Errico J.M., Theel E.S., Liebeskind M.J. (2021). Identification of SARS-CoV-2 spike mutations that attenuate monoclonal and serum antibody neutralization. Cell Host Microbe.

[13] Thomson E.C., Rosen L.E., Shepherd J.G., Spreafico R., da Silva Filipe A., Wojcechowskyj J.A., Davis C., Piccoli L., Pascall D.J., Dillen J. (2021). Circulating SARS-CoV-2 spike N439K variants maintain fitness while evading antibody-mediated immunity. Cell.

[14] Ali A., Vijayan R. (2020). Dynamics of the ACE2–SARS-CoV-2/SARS-CoV spike protein interface reveal unique mechanisms. Sci. Rep..

[15] Bai C., Warshel A. (2020). Critical Differences between the Binding Features of the Spike proteins of SARS-CoV-2 and SARS-CoV. J. Phys. Chem. B.

[16] Laurini E., Marson D., Aulic S., Fermeglia M., Pricl S. (2020). Computational Alanine Scanning and Structural Analysis of the SARS-CoV-2 Spike Protein/Angiotensin-Converting Enzyme 2 Complex. ACS Nano.

[17] Bai C., Wang J., Chen G., Zhang H., An K., Xu P., Du Y., Ye R.D., Saha A., Zhang A. (2021). Predicting Mutational Effects on Receptor Binding of the Spike Protein of SARS-CoV-2 Variants. J. Am. Chem. Soc..

[18] Verkhivker G., Agajanian S., Kassab R., Krishnan K. (2022). Computer Simulations and Network-Based Profiling of Binding and Allosteric Interactions of SARS-CoV-2 Spike Variant Complexes and the Host Receptor: Dissecting the Mechanistic Effects of the Delta and Omicron Mutations. Int. J. Mol. Sci..

[19] Pitsillou E., Liang J.J., Beh R.C., Hung A., Karagiannis T.C. (2022). Molecular dynamics simulations highlight the altered binding landscape at the spike-ACE2 interface between the Delta and Omicron variants compared to the SARS-CoV-2 original strain. Comput. Biol. Med..

[20] Khan A., Khan S.A., Zia K., Altowyan M.S., Barakat A., Ul-Haq Z. (2022). Deciphering the Impact of Mutations on the Binding Efficacy of SARS-CoV-2 Omicron and Delta Variants With Human ACE2 Receptor. Front. Chem..

[21] An K., Yang X., Luo M., Yan J., Xu P., Zhang H., Li Y., Wu S., Warshel A., Bai C. (2024). Mechanistic study of the transmission pattern of the SARS-CoV-2 omicron variant. Proteins.

[22] Kumar R., Murugan N.A., Srivastava V. (2022). Improved Binding Affinity of Omicron’s Spike Protein for the Human Angiotensin-Converting Enzyme 2 Receptor Is the Key behind Its Increased Virulence. Int. J. Mol. Sci..

[23] Verkhivker G.M., Di Paola L. (2021). Integrated Biophysical Modeling of the SARS-CoV-2 Spike Protein Binding and Allosteric Interactions with Antibodies. J. Phys. Chem. B.

[24] da Costa C.H., de Freitas C.A., Alves C.N., Lameira J. (2022). Assessment of mutations on RBD in the Spike protein of SARS-CoV-2 Alpha, Delta and Omicron variants. Sci. Rep..

[25] Jawad B., Adhikari P., Podgornik R., Ching W.Y. (2021). Key Interacting Residues between RBD of SARS-CoV2 and ACE2 Receptor: Combination of Molecular Dynamics Simulation and Density Functional Calculation. J. Chem. Inf. Model..

[26] Jawad B., Adhikari P., Podgornik R., Ching W.Y. (2022). Binding Interactions between Receptor-Binding Domain of Spike Protein and Human Angiotensin Converting Enzyme-2 in Omicron Variant. J. Phys. Chem. Lett..

[27] Gumbart J.C., Roux B., Chipot C. (2013). Efficient determination of protein-protein standard binding free energies from first principles. J. Chem. Theory Comput..

[28] Sun Z., Yan Y.N., Yang M., Zhang J.Z. (2017). Interaction entropy for protein-protein binding. J. Chem. Phys..

[29] Lan J., Ge J., Yu J., Shan S., Zhou H., Fan S., Zhang Q., Shi X., Wang Q., Zhang L. (2020). Structure of the SARS-CoV-2 spike receptor-binding domain bound to the ACE2 receptor. Nature.

[30] Saville J.W., Mannar D., Zhu X., Srivastava S.S., Berezuk A.M., Demers J.P., Zhou S., Tuttle K.S., Sekirov I., Kim A. (2022). Structural and biochemical rationale for enhanced spike protein fitness in delta and kappa SARS-CoV-2 variants. Nat. Commun..

[31] Geng Q., Shi K., Ye G., Zhang W., Aihara H., Li F. (2022). Structural Basis for Human Receptor Recognition by SARS-CoV-2 Omicron. Variant BA.1. J. Virol..

[32] Yin W., Xu Y., Xu P., Cao X., Wu C., Gu C., He X., Wang X., Huang S., Yuan Q. (2022). Structures of the Omicron spike trimer with ACE2 and an anti-Omicron antibody. Science.

[33] Kastritis P.L., Bonvin A.M.J.J. (2013). On the binding affinity of macromolecular interactions: Daring to ask why proteins interact. J. R. Soc. Interface.

[34] Krissinel E., Henrick K. (2007). Inference of Macromolecular Assemblies from the Crystalline State. J. Mol. Biol..

[35] He W., Liu X., Hu B., Li D., Chen L., Li Y., Tu Y., Xiong S., Wang G., Deng J. (2022). Mechanisms of SARS-CoV-2 Infection-Induced Kidney Injury: A Literature Review. Front. Cell. Infect. Microbiol..

[36] Meng B., Abdullahi A., Ferreira I.A., Goonawardane N., Saito A., Kimura I., Yamasoba D., Gerber P.P., Fatihi S., Rathore S. (2022). Altered TMPRSS2 usage by SARS-CoV-2 Omicron impacts infectivity and fusogenicity. Nature.

[37] Padmanabhan P., Dixit N.M. (2023). Modelling how increased Cathepsin B/L and decreased TMPRSS2 usage for cell entry by the SARS-CoV-2 Omicron variant may affect the efficacy and synergy of TMPRSS2 and Cathepsin B/L inhibitors. J. Theo. Biol..

[38] Jo S., Kim T., Iyer V.G., Im W. (2008). CHARMM-GUI: A web-based graphical user interface for CHARMM. J. Comput. Chem..

[39] Brooks B.R., Brooks C.L., Mackerell A.D., Nilsson L., Petrella R.J., Roux B., Won Y., Archontis G., Bartels C., Boresch S. (2009). CHARMM: The biomolecular simulation program. J. Comput. Chem..

[40] Im W., Lee M.S., Brooks C.L. (2003). Generalized Born Model with a Simple Smoothing Function. J. Comput. Chem..

[41] Pastor R.W., Luckhurst G.R., Veracini C.A. (1994). Techniques and Applications of Langevin Dynamics Simulations. The Molecular Dynamics of Liquid Crystals.

[42] BIOVIA Dassault Systèmes (2018). Discovery Studio Visualizer.

[43] Chen Y., Zhao X., Zhou H., Zhu H., Jiang S., Wang P. (2023). Broadly neutralizing antibodies to SARS-CoV-2 and other human coronaviruses. Nat. Rev. Immunol..

